# Bohlmann-Rahtz Cyclodehydration of Aminodienones to Pyridines Using *N*-Iodosuccinimide 

**DOI:** 10.3390/molecules15053211

**Published:** 2010-04-30

**Authors:** Mark C. Bagley, Christian Glover

**Affiliations:** School of Chemistry, Main Building, Cardiff University, Park Place, Cardiff, CF10 3AT, UK

**Keywords:** Bohlmann-Rahtz synthesis, cyclization reactions, heterocycles, Lewis acids, pyridines

## Abstract

Cyclodehydration of Bohlmann-Rahtz aminodienone intermediates using *N*‑iodosuccinimide as a Lewis acid proceeds at low temperature under very mild conditions to give the corresponding 2,3,6-trisubstituted pyridines in high yield and with total regiocontrol.

## 1. Introduction

**Scheme 1:**
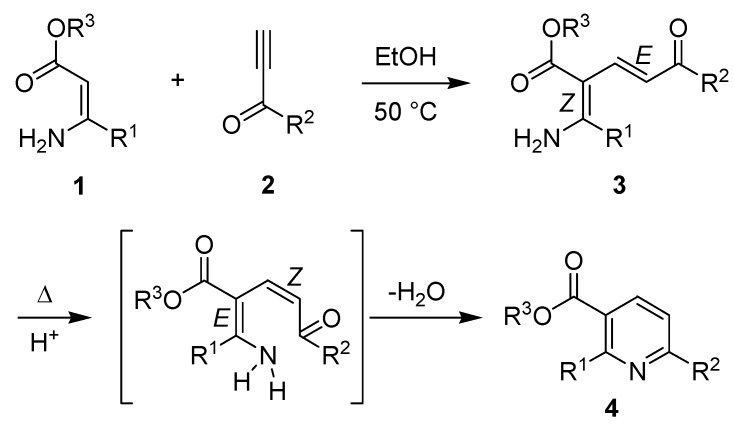
Two-step Bohlmann-Rahtz synthesis of 2,3,6-trisubstituted pyridines **4**.

The synthesis, reactions and properties of pyridine-containing derivatives is an important component of modern heterocyclic chemistry. This heterocyclic motif is found in a large number of pharmaceutical agents [1], as a pharmacophore of considerable importance, and a valuable synthetic building block in drug discovery, heterocyclic chemistry and natural product synthesis [2]. Thus, new and facile methods for the synthesis of polysubstituted pyridines are of considerable current interest, in particular if they have a predictable and reliable regiochemical outcome and provide the heterocycle in high yield from readily-available precursors. The Bohlmann-Rahtz pyridine synthesis bears close relation to many well-established approaches to pyridines based upon heterocyclocondensation processes, such as the Hantzsch dihydropyridine synthesis [3], and was first reported back in 1957 [4]. This robust two-step method provides an efficient route to 2,3,6-trisubstituted pyridines **4** from stabilized enamines **1** and ethynyl ketones **2**. It proceeds by an initial Michael addition to give an aminodienone intermediate **3**, which is isolated and purified, and subsequent cyclodehydration under forcing conditions (up to 200 ºC) in order to facilitate *E*/*Z*-isomerization and subsequent spontaneous heterocyclization to the pyridine product **4 **(Scheme 1). Since its discovery, this heterocyclocondensation reaction has received very little attention until recently [5], when it has found application in the synthesis of pyridine libraries [6,7], pyrido[2,3-*d*]pyrimidines [8,9,10,11], α-helix mimetics [12], nicotinonitrile-derived chromophores with tunable photophysical properties [13,14], heterocyclic amino acids [15,16,17], and in the preparation of pyridine-containing natural products such as the thiopeptide antibiotics [18,19,20,21,22] and chemical derivatives thereof [5,23,24,25,26,27].

Given its wide range of potential applications, many efforts have been made to improve the Bohlmann-Rahtz pyridine synthesis, with particular emphasis on catalyzing the *E*/*Z*-isomerization and subsequent cyclodehydration in order to avoid the use of very high temperatures and broaden its substrate specificity. Recent findings have shown that the heterocyclization can be accelerated through the use of a Brønsted or Lewis acid [5,9,28,29] and can be carried out in just one operation through the use of a protic solvent [30] or at high temperature under microwave dielectric heating [31]. Alternative processing methods have also been reported for the cyclodehydration reaction, such the use of continuous flow reactors [32,33], and this transformation can be incorporated into a one-pot multistep process by combining with reactions such as tandem oxidation [34] or enamine formation [35] which improves the overall chemical yield. However, despite the synthetic utility of Bohlmann-Rahtz aminodienones **3**, as evidenced by their widespread application [5,6,7,8,9,10,11,12,13,14,15,16,17,18,19,20,21,22,23,24,25,26,27], no studies had been undertaken to divert these intermediates down an alternative reaction path in order to expand the scope of possible products.

**Scheme 2:**
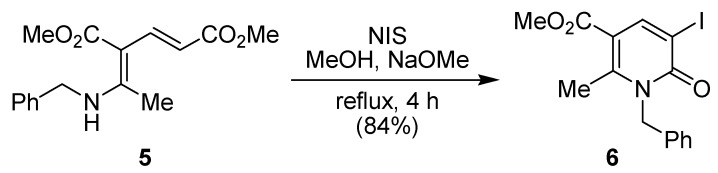
Synthesis of 3-iodo-2(1*H*)-pyridinone **6** from δ-dienaminoester **5** by Dechoux and co-workers [36].

In 2002, Dechoux and co-workers described that the δ-dienaminoester **5** reacted with *N*‑iodosuccinimide (NIS) in methanol in the presence of sodium methoxide to give the corresponding 3-iodo-2(1*H*)-pyridinone **6** in 84% yield (Scheme 2) [36]. 

**Scheme 3:**
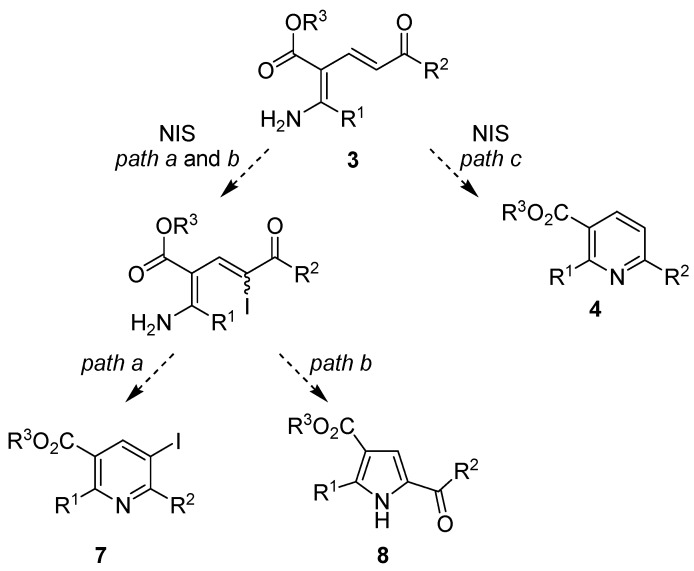
Possible mechanistic course (*path a*, *b* or *c*) of the reaction between Bohlmann-Rahtzaminodienones **3** and NIS.

The reaction was found to be highly chemoselective and yet could be diverted to provide the polysubstituted pyrrole as an alternative product simply by changing the reagent and altering the conditions [37]. The use of NIS as an electrophilic iodinating agent for aldehydes, ketones and alkenes is well reported but recent developments have seen a host of further applications, such as the use of Brønsted or Lewis acids to improve its reactivity in electrophilic aromatic substitution reactions [38,39,40], asymmetric iodination of aldehydes using an axially chiral catalyst [41] and 1,3-dicarbonyl compounds under mild conditions [42], synthesis of haloalkenes and haloalkynes by catalytic Hunsdiecker reaction [43], the reaction of alkynes and NIS/water to give α-diketones [44], highly enantioselective iodocyclization of polyprenoids [45] and Au-catalyzed formation of 2-iodoenones from propargylic alcohol derivatives [46,47], amongst others. Interestingly, there have also been a few reports on the use of NIS as a mild and selective Lewis acid, such as in the catalytic deprotection of TBDMS ethers to alcohols in methanol under ambient conditions [48] and the *N*-debenzylation of benzylamino alcohols [49]. Given this spectrum of reactivity and the precedent offered by Dechoux [36,37], we set out to examine the reaction of the Bohlmann-Rahtz aminodienone intermediate **3** with NIS to establish the mechanistic course (Scheme 3). It was anticipated that reaction could proceed by iodination followed by heteroannulation to give the 5-iodopyridine **7 **in a similar fashion to Dechoux’s pyridinone synthesis [36] (*path a*), iodination followed by displacement of iodide to give the acylpyrrole **8** following Dechoux’s pyrrole-5-carboxylate synthesis [37] (*path b*), or whether it would follow a new reaction course as a Lewis acid to give the Bohlmann-Rahtz pyridine **4** (*path c*).

## 2. Results and Discussion

**Scheme 4:**
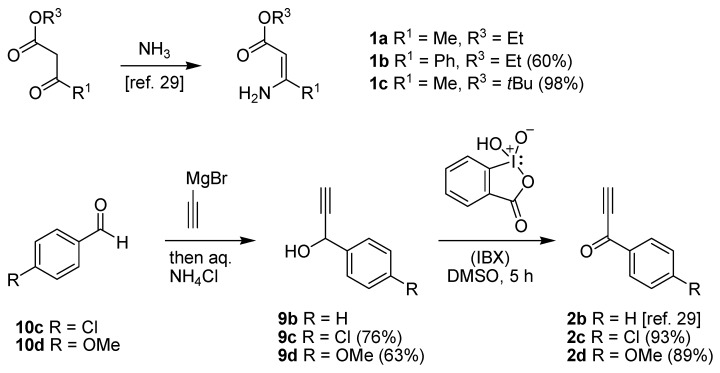
Enamine and aryl ethynylketone subsets, **1a-c **and **2b-d**, respectively.

In order to test the behaviour of the Bohlmann-Rahtz intermediates on treatment with NIS, a sample library of aminodienones **3** was prepared from the corresponding enamines **1** and ethynyl ketones **2**. The enamine subset **1a-c** was generated according to our previously reported procedure (Scheme 4) [29] whereas the ethynyl ketone subset employed 4-(trimethylsilyl)but-3-yn-2-one **2a** as a but-3-yn-2-one surrogate [29] and was complemented by additional aryl ethynyl ketone subset members **2b-d** prepared by the *o*-iodoxybenzoic acid (IBX)-mediated oxidation [51] of propargylic alcohols **9b-d**, which in turn could be generated by addition of ethynylmagnesium bromide to the corresponding benzaldehyde **10c,d **[13,29,50] (Figure 4). This small library of ethynyl ketones **2a-d** was chosen to probe if electronic effects had a major influence upon the mechanistic course and, while certainly not exhaustive in scope, was felt to be representative. 

The Michael addition of the enamine subset **1a-c** and ethynyl ketones **2a-d** was carried out under traditional Bohlmann-Rahtz conditions in ethanol at 50 ºC to give pure aminodienones **3 **after purification by column chromatography on silica, in order to ensure no contamination from the corresponding pyridines **4** was evident prior to treatment with NIS. For the most part, the Michael addition reactions were highly efficient, apart from the synthesis of phenyldienone **3ba**, which was prepared in low yield as significant cyclodehydration to **4ba** (which was removed on purification) occurred spontaneously under the reaction conditions (Table 1). 

With pure samples of the Bohlmann-Rahtz aminodienone intermediates **3** in hand, each was treated in turn with a stoichiometric quantity of NIS in ethanol at 0 ºC. In all cases, including both electron-poor and electron-rich aryldienones, only one reaction course was seen to operate: NIS was found to behave as a Lewis acid under these conditions, promoting spontaneous cyclodehydration of the normally kinetically-stable aminodienones to give the corresponding pyridine **4** in excellent yield (Table 1). In all cases only a single pyridine regioisomer was obtained, in accordance with the course of a Bohlmann-Rahtz cyclodehydration reaction [4,5]. The only substrate that was not efficient in this process was aminoheptadienone **3aa** (entry 1), but by extending the reaction time to 4 h, pyridine **4aa** was isolated in an acceptable 84% yield. All of the other substrates gave a near quantitative yield of the corresponding pyridine products **4**. Furthermore, in order to establish the role of the NIS, one of these cyclodehydration reactions were carried out in the presence of a catalytic quantity (20 mol % NIS) of reagent (entry 3) and, for this substrate, the final yield of pyridine **4ab** was found to be unchanged (>98% isolated yield).

**Table 1:** Synthesis of aminodienones**3** and their cyclodehydration to pyridines **4** mediated by NIS.

Entry	1	2	3	Yield% *^a^*	4	Yield% *^b^*
1	**1a**	**2a**	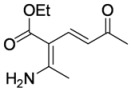	**3aa** (82)	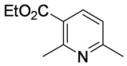	**4aa** (66 or 84*^c^*)
2	**1b**	**2a**	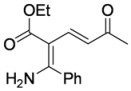	**3ba** (23)	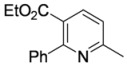	**4ba** (>98)
3	**1a**	**2b**	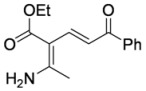	**3ab** (85)	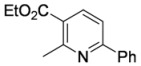	**4ab** (>98)*^d^*
4	**1a**	**2c**	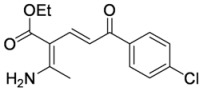	**3ac** (86)	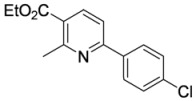	**4ac** (97)
5	**1a**	**2d**	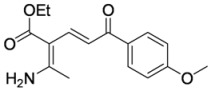	**3ad** (95)	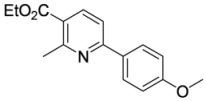	**4ad** (>98)
6	**1c**	**2a**	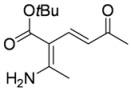	**3ca** (68)	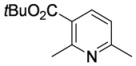	**4ca** (>98)*^e^*

*^a^* Isolated yield of **3**, obtained after heating a solution of the corresponding subset members **1** and **2** in EtOH at 50 ºC followed by chromatographic purification on silica gel, is given in parentheses; *^b^* Isolated yield of **4**, obtained after treatment with NIS (1 equiv.) in EtOH at 0 ºC followed by chromatographic purification on silica gel, is given in parentheses; *^c^* Reaction with NIS was carried out over 4 h at 0 ºC; *^d^* Isolated yield of **4ab** was unchanged using either a stoichiometric (1 equiv.) or catalytic (20 mol%) amount of NIS; *^e^* Isolated yield of **4ca** was unchanged if the reaction was carried out in the presence of NaHCO_3_.

The reaction course followed by Bohlmann-Rahtz intermediates on treatment with NIS (Scheme 3, *path c*) is in contrast to the behaviour of aminodienoates such as **5** as reported by Dechoux, which give the corresponding pyrrole derivatives under comparable reaction conditions (in analogy to Scheme 3, *path b*). This contrasting behaviour could be attributed to the increased reactivity of the ketone towards cyclodehydration but nonetheless is a remarkable switch in mechanism. The surprising facility of this process is also worthy of note. Typically, the Bohlmann-Rahtz cyclodehydration requires high temperatures (up to 200 ºC) or the action of a Brønsted or Lewis acid under heating (microwave or conventional), but, for most of the substrates examined, a near quantitative yield was obtained after only 1 h at 0 ºC. In order to establish if traces of a Brønsted acid (such as HI) had been responsible for the extremely facile cyclodehydration, the reaction of aminodienone **3ca** with NIS (1 equiv.) was repeated in the presence of NaHCO_3_ base (Table 1, entry 6), but the yield of pyridine **4ca** was found to be unchanged (>98% isolated yield). Purification of the NIS by recrystallization immediately prior to use was also found to have no effect upon the reaction course or efficiency. Furthermore, in the absence of NIS, when aminodienone **3aa** was stirred in EtOH at 0 ºC, no cyclodehydration occurred at all and only unreacted starting material was obtained, as identified by ^1^H-NMR spectroscopic analysis, confirming a catalytic role for the reagent in the *Z/E* isomerisation and subsequent spontaneous cyclodehydration. 

## 3. Experimental Section

### 3.1. General

Commercially available reagents were used without further purification; solvents were dried by standard procedures. Light petroleum refers to the fraction with bp 40–60 ºC, ether (Et_2_O) refers to diethyl ether and EtOAc refers to ethyl acetate. Column chromatography was carried out using Merck Kieselgel 60 H silica or Matrex silica 60. Analytical thin layer chromatography was carried out using aluminium-backed plates coated with Merck Kieselgel 60 GF_254_ that were visualised under UV light (at 254 and/or 360 nm). Melting points (mp) were determined on a Kofler hot stage apparatus and are uncorrected. Infra–red (IR) spectra were recorded in the range 4,000–600 cm^–1^ on a Perkin-Elmer 1600 series FTIR spectrometer using KBr disks for solid samples and thin films between NaCl plates for liquid samples or as a nujol mull and are reported in cm^–1^. Nuclear magnetic resonance (NMR) spectra were recorded in CDCl_3_ at 25 ºC unless stated otherwise using a Bruker DPX 400 or 500 Avance instrument operating at 400 or 500 MHz for ^1^H spectra and 100 or 125 MHz for ^13^C spectra and were reported in ppm; *J *values were recorded in Hz and multiplicities were expressed by the usual conventions (s = singlet, d = doublet, t = triplet, app = apparent, m = multiplet). Low-resolution mass spectra (MS) were determined using a Fisons VG Platform II Quadrupole instrument using atmospheric pressure chemical ionization (APcI) unless stated otherwise. ES refers to electrospray ionization, CI refers to chemical ionization (ammonia) and EI refers to electron impact. High-resolution mass spectra were obtained courtesy of the EPSRC Mass Spectrometry Service at Swansea, UK using the ionisation methods specified. Microanalyses were recorded using a Perkin-Elmer 240C Elemental Analyzer. *In vacuo* refers to evaporation at reduced pressure using a rotary evaporator and diaphragm pump, followed by the removal of trace volatiles using a vacuum (oil) pump. 

### 3.2. Typical experimental procedures

#### 3.2.1. Typical procedure for Michael addition of an enamine **1** and ethynyl ketone **2**

A solution of the enamine **1** (0.36 mmol, 1 equiv.) and alkynone **2** (0.56 mmol, 1.5 equiv.) in EtOH (5 mL) was stirred at 50 ºC for 1–7 h, cooled and evaporated *in vacuo* to give the crude product. Purification by column chromatography on silica gel, eluting with EtOAc–light petroleum, gave the aminodienone **3**.

#### 3.2.2. Typical procedure for cyclodehydration of a Bohlmann-Rahtz aminodienone **3** using NIS

A solution of the aminodienone **3** (0.2 mmol, 1 equiv.) and *N*-iodosuccinimide (NIS) (0.25 mmol, 1.2 equiv.) in EtOH (4 mL) was stirred at 0 ºC for 1 h and then evaporated *in vacuo* to give the crude product. Purification by column chromatography on silica gel, eluting with EtOAc–light petroleum, gave the pyridine **4**.

#### 3.2.3. Typical procedure for the synthesis of propargylic alcohols **9** from aldehydes **10**

A solution of the aldehyde **10** (5.0 mmol) in dry THF (10 mL) was added to a stirred solution of ethynylmagnesium bromide in THF (0.5 M; 15 mL, 7.5 mmol) at 0 ºC. The mixture was stirred at 0 ºC for 2 h, warmed to room temperature and stirred overnight. Saturated aqueous NH_4_Cl solution (2 mL) was added. The mixture was evaporated *in vacuo* and partitioned between ether (30 mL) and saturated aqueous ammonium chloride solution (30 mL). The ethereal layer was washed with brine (30 mL), dried (Na_2_SO_4_) and evaporated *in vacuo*.

### 3.3. Experimental procedures

#### 3.3.1. Ethyl 3-amino-3-phenylpropenoate (**1b**)

Ammonium acetate (13.4 g, 0.17 mol) was added to a solution of ethyl benzoylacetate (5 mL, 29.0 mmol) and the mixture was heated at reflux in toluene–glacial acetic acid (5:1; 40 mL) for 20 h. After partitioning between H_2_O (100 mL) and ether (60 mL), the aqueous layer was further extracted with ether (2 × 25 mL) and the combined organic extracts were washed sequentially with saturated aqueous NaHCO_3_ solution (50 mL) and brine (25 mL), dried (MgSO_4_) and evaporated *in vacuo*. Purification by column chromatography on SiO_2_ gel, eluting with light petroleum–EtOAc (3:1) (*R_f_* = 0.11), gave the title compound [29] as a pale yellow oil (3.32 g, 60%) (Found: MNH_4_^+^, 209.1289. C_11_H_17_N_2_O_2_ [*MNH_4_^+^*] requires 209.1285); IR (film)/cm^-1^* ν_max_* 3,441, 3,326, 2,979, 2,936, 1,663, 1,617, 1,555, 1,492, 1,364, 1,176, 1,095, 1,025, 796, 772, 699; ^1^H-NMR (400 MHz; CDCl_3_) *δ_H_* 8.35 (1H, bs, NH), 7.33–7.12 (5H), 7.00 (1H, bs, NH), 4.75 (1H, s, CH), 3.95 (2H, q, *J* = 7.1 Hz, OC*H*_2_Me), 1.05 (3H, t, *J* = 7.1 Hz, CH_2_*Me*); ^13^C-NMR (100 MHz, CDCl_3_) *δ_C_* 170.5 (C), 160.5 (C), 137.7 (C), 130.3 (CH), 128.9 (CH), 126.2 (CH), 84.7 (CH), 59.0 (CH_2_), 14.6 (Me); *m/z* (APcI) 192 (MH^+^, 100%) and 146 (13).

#### 3.3.2. tert-Butyl β-aminocrotonate (**1c**)

Ammonium hydroxide solution (35%, 40 mL) was added to *tert*-butyl acetoacetate (4 mL, 24.2 mmol) in MeOH (40 mL) and the mixture was stirred at 50 ºC for 18 h. After cooling, the solution was evaporated *in vacuo* and partitioned between H_2_O (40 mL) and ether (40 mL). The aqueous layer was further extracted with EtOAc (2 × 35 mL) and the combined organic extracts were washed with brine (25 mL), dried (Na_2_SO_4_) and evaporated *in vacuo* to give the title compound [52] as a colourless oil (3.72 g, 98%) (Found: MH^+^, 158.1178. C_8_H_16_NO_2_ [*MH^+^*] requires 158.1176); IR (film)/cm^-1^ *ν_max_* 3,554, 3,341, 2,980, 2,919, 1,666, 1,622, 1,567, 1,454, 1,390, 1,366, 1,296, 1,150, 983, 790; ^1^H-NMR (400 MHz; CDCl_3_) *δ_H_* 8.20 (1H, bs, NH), 4.20 (1H, bs, NH), 4.35 (1H, s, CH), 1.80 (3H, s, Me), 1.38 (9H, s, CMe_3_); ^13^C-NMR (100 MHz, CDCl_3_) *δ_C_* 170.3 (C), 158.8 (C), 85.9 (CH), 78.2 (C), 28.6 (Me), 22.4 (Me); *m/z* (APcI) 158 (MH^+^, 77%).

#### 3.3.3. 1-Phenylprop-2-yn-1-one (**2b**)

A solution of *o-*iodoxybenzoic acid (IBX) [51] (3.65 g, 13.0 mmol) in DMSO (110 mL) was stirred for 15 min at room temperature until homogeneous. A solution of 1-phenyl-2-propyn-1-ol **9b **(1.32 g, 10.0 mmol) in DMSO (10 mL) was added and the mixture was stirred for 5 h. H_2_O (30 mL) was added and the mixture was stirred at room temperature for 10 min, cooled in ice and partitioned between H_2_O (120 mL) and ether (90 mL). The mixture was filtered through Celite^®^ and the aqueous layer was further extracted with ether (50 mL). The organic extracts were combined, washed sequentially with H_2_O (3 × 50 mL), saturated aqueous NaHCO_3_ solution (70 mL) and brine (70 mL), dried (Na_2_SO_4_) and evaporated *in vacuo* to give the title compound as a pale yellow solid (1.0 g, 77%), m.p. 49–50 ºC (MeOH) (literature [29] m.p. 47–48 ºC) (Found: M^+^, 130.0414. C_9_H_6_O [*M^+^*] requires 130.0413); IR (KBr)/cm^-1^ *ν_max_* 3,231, 2,094, 1,645, 1,593, 1,578, 1,452, 1,317, 1,261, 1,173, 1,005, 695; ^1^H-NMR (400 MHz; CDCl_3_) *δ_H_* 8.12 (2H, m, *o*-Ph*H*), 7.55 (1H, m, *p*-Ph*H*), 7.45 (2H, m, *m*-Ph*H*), 3.36 (1H, s, CH); ^13^C-NMR (100 MHz; CDCl_3_) *δ_C_* 177.5 (C), 136.1 (C), 134.6 (CH), 129.7 (CH), 128.7 (CH), 80.9 (C), 80.3 (CH); *m/z* (EI) 130 (M^•+^, 16%), 77 (32), 53 (100).

#### 3.3.4. 1-(4-Chlorophenyl)prop-2-yn-1-one (**2c**)

Propargylic alcohol **9c** was prepared using aldehyde **10c** according to the general procedure 3.2.3. Purification by column chromatography on SiO_2_ gel, eluting with CH_2_Cl_2_ (*R_f_* 0.27), gave *1-(4-chlorophenyl)prop-2-yn-1-ol* (**9c**) [50] as a pale yellow oil (635 mg, 76%) (Found: M^+^, 166.0181. C_9_H_7_ClO [*M^+^*] requires 166.0180); IR (film)/cm^–1^*ν_max_* 3418, 3296, 2884, 2119, 1904, 1645, 1597, 1490, 1406, 1257, 1192, 1092, 1015, 950, 909, 835, 791, 734, 650; ^1^H NMR (400 MHz; CDCl_3_) *δ_H_* 7.48 (2H, app d, *J* = 8.4 Hz, 2’,6’-PhH), 7.33 (2H, app d, *J* = 8.4 Hz, 3’,5’-PhH), 5.45 (1H, s, 1-H), 2.68 (1H, s, 3-H), 2.28 (1H, s, OH); ^13^C NMR (100 MHz; CDCl_3_) *δ_C_* 138.4 (C), 134.4 (C), 128.8 (CH), 128.0 (CH), 83.1 (CH), 75.3 (C), 63.7 (CH); *m/z* (EI) 166 (M^•+^, 9%), 164 (27), 113 (5), 111 (14), 53 (100). A solution of IBX (5.84 g, 20.8 mmol) in DMSO (120 mL) was stirred for 15 min at room temperature until homogeneous. A solution of 1-(4-chlorophenyl)prop-2-yn-1-ol (**9c**) (2.72 g, 16.3 mmol) in DMSO (20 mL) was added and the mixture was stirred for 5 h. H_2_O (40 mL) was added and the mixture was stirred at room temperature for 10 min, cooled in ice and partitioned between H_2_O (120 mL) and ether (90 mL). The mixture was filtered through Celite^®^ and the aqueous layer was further extracted with ether (50 mL). The organic extracts were combined, washed sequentially with H_2_O (3 × 50 mL), saturated aqueous NaHCO_3_ solution (70 mL) and brine (70 mL), dried (Na_2_SO_4_) and evaporated *in vacuo*. Purification by column chromatography on SiO_2_ gel, eluting with CHCl_3_ (*R_f_* = 0.45), gave the title compound as a pale yellow solid (2.50 g, 93%), m.p. 68–69 ºC (MeOH) (literature [6] m.p. 68–69 ºC) (Found: M^+^, 165.9991. C_9_H_5_Cl^37^O [*M^+^*] requires 165.9994); IR (KBr)/cm^-1^ *ν_max_* 2,921, 2,853, 2,360, 1,662, 1,462, 1,377, 1,248, 1,094, 1,003, 722; ^1^H-NMR (400 MHz; CDCl_3_) *δ_H_* 7.98 (2H, app d, *J* = 8.6 Hz, 2’,6’-PhH), 7.41 (2H, app d, *J* = 8.6 Hz, 3’,5’-PhH), 3.38 (1H, s, CH); ^13^C-NMR (100 MHz; CDCl_3_) *δ_C_* 176.1 (C), 141.3 (C), 134.5 (C), 131.0 (CH), 129.1 (CH), 81.4 (C), 79.9 (CH); *m/z* (EI) 166 (M^•+^, 7%), 164 (M^•+^, 21), 138 (11), 136 (33), 113 (3), 111 (12), 53 (100).

#### 3.3.5. 1-(4-Methoxyphenyl)prop-2-yn-1-one (**2d**)

Propargylic alcohol **9d** was prepared using aldehyde **10d** according to the general procedure 3.2.3. Purification by column chromatography on SiO_2_ gel, eluting with CH_2_Cl_2_ (*R_f_* 0.10), gave *1-(4-methoxyphenyl)prop-2-yn-1-ol* (**9c**) [6,53] as a pale yellow oil (513 mg, 63%) (Found: MH^+^, 161.0596. C_10_H_10_O_2_ [*MH^+^*] requires 161.0597); IR (film)/cm^-1^ *ν_max_* 3,438, 3,284, 3,003, 2,935, 2,837, 1,892, 1,611, 1,512, 1,464, 1,442, 1,304, 1,249, 1,174, 1,112, 1,032, 948, 833, 768; ^1^H-NMR (400 MHz; CDCl_3_) *δ_H_* 7.50 (2H, app d, *J* 8.6, 2’,6’-PhH), 6.90 (2H, app d, *J* 8.6, 3’,5’-PhH), 5.42 (1H, s, 1-H), 3.85 (3H, s, OMe), 2.65 (1H, s, 3-H); ^13^C-NMR (100 MHz; CDCl_3_) *δ_C_* 159.8 (C), 132.4 (C), 128.1 (CH), 114.0 (CH), 83.7 (CH), 74.7 (C), 64.0 (CH), 55.4 (Me); *m/z* (EI) 162 (M^•+^, 100%), 161 (54), 145 (35), 131 (38), 89 (57), 53 (43). A solution of IBX (5.14 g, 18.3 mmol) in DMSO (120 mL) was stirred for 15 min at room temperature until homogeneous. A solution of 1-(4-methoxyphenyl)prop-2-yn-1-ol (**9d**) (2.27 g, 14.0 mmol) in DMSO (20 mL) was added and the mixture was stirred for 5 h. H_2_O (40 mL) was added and the mixture was stirred at room temperature for 10 min, cooled in ice and partitioned between H_2_O (120 mL) and ether (90 mL). The mixture was filtered through Celite^®^ and the aqueous layer was further extracted with ether (80 mL). The organic extracts were combined, washed sequentially with H_2_O (3 × 50 mL), saturated aqueous NaHCO_3_ solution (70 mL) and brine (70 mL), dried (Na_2_SO_4_) and evaporated *in vacuo*. Purification by column chromatography on SiO_2_ gel, eluting with CHCl_3_ (*R_f_* = 0.29), gave the title compound as a pale yellow solid (2.50 g, 93%), m.p. 86–87 ºC (MeOH) (literature [6] m.p. 85–87 ºC) (Found: MH^+^, 161.0597. C_10_H_9_O_2_ [*MH^+^*] requires 161.0597); IR (KBr)/cm^-1^ *ν_max_* 3,297, 2,092, 1,641, 1,597, 1,572, 1,511, 1,423, 1,252, 1,170, 1,116, 1,023, 841, 758, 710, 685; ^1^H-NMR (400 MHz; CDCl_3_) *δ_H_* 8.05 (2H, app d, *J* = 8.7 Hz, 2’,6-PhH), 6.88 (2H, app d, *J* = 8.7 Hz, 3’,5’-PhH), 3.88 (3H, s, OMe), 3.29 (1H, s, CH); ^13^C-NMR (100 MHz; CDCl_3_) *δ_C_* 176.0 (C), 164.8 (C), 132.2 (CH), 129.2 (CH), 113.9 (CH), 80.4 (C), 80.1 (C), 55.6 (Me); *m/z* (APcI) 161 (MH^+^, 100%).

#### 3.3.6. (2Z,4E)-2-Amino-3-ethoxycarbonylheptadien-6-one (**3aa**)

Aminodienone **3aa** was prepared according to the general procedure 3.2.1 using ethyl β-aminocrotonate (**1a**) and 4-(trimethylsilyl)but-3-yn-2-one (**2a**). Purification by column chromatography on SiO_2_ gel, eluting with light petroleum–EtOAc (1:1) (*R_f_* 0.23), gave the title compound as a yellow solid (58 mg, 82%), m.p. 125–126 ºC(light petroleum–EtOAc) (literature [29] m.p. 125.5–126.4 ºC) (Found: C, 60.6; H, 7.7; N, 7.0. Calc. for C_10_H_15_NO_3_: C, 60.1; H, 7.7; N, 7.1%) (Found: MH^+^, 198.1125. C_10_H_16_NO_3_ [*MH^+^*] requires 198.1125); IR (KBr)/cm^-1^ *ν_max_* 3,334, 3,193, 2,977, 1,647, 1,546, 1,488, 1,459, 1,362, 1,319, 1,286, 1,205, 1,180, 1,112, 1,024, 970, 950, 844; ^1^H- NMR (400 MHz; CDCl_3_) *δ_H_* 9.65 (1H, bs, NH), 7.55 (1H, d, *J* = 15.6 Hz, 4-H), 6.50 (1H, d, *J* = 15.6 Hz, 5-H), 5.5 (1H, bs, NH), 4.22 (2H, q, *J* = 7.1 Hz, OC*H*_2_Me), 2.22 (3H, s, Me), 2.15 (3H, s, Me), 1.28 (3H, t, *J* = 7.1 Hz, CH_2_*Me*); ^13^C-NMR (100 MHz; CDCl_3_) *δ_C_* 199.0 (C), 169.7 (C), 165.7 (C), 139.5 (CH), 121.1 (CH), 94.4 (C), 60.0 (CH_2_), 28.4 (Me), 22.6 (Me), 14.4 (Me); *m/z* (APcI) 198 (MH^+^, 100%), 181 (48).

#### 3.3.7. (2Z,4E)-2-Amino-3-ethoxycarbonyl-1-phenylhexadien-6-one (**3ba**)

Aminodienone **3ba** was prepared according to the general procedure 3.2.1 using ethyl 3-amino-3-phenylpropenoate (**1b**) and 4-(trimethylsilyl)but-3-yn-2-one (**2a**). Purification by column chromatography on SiO_2_ gel, eluting with light petroleum–EtOAc (1:1) (*R_f_* 0.24), gave the title compound as a yellow solid (22 mg, 23%), m.p. 104–105 ºC (light petroleum–EtOAc) (Found: C, 69.2; H, 6.6; N, 5.2. Calc. for C_15_H_17_NO_3_: C, 69.5; H, 6.6; N, 5.4%); IR (KBr)/cm^-1^ *ν_max_* 3,317, 3,120, 2,980, 1,654, 1,586, 1,507, 1,461, 1,345, 1,285, 1,257, 1,210, 1,119, 1,023, 1,002, 979, 927, 852, 768, 702, 632, 358; ^1^H-NMR (400 MHz; CDCl_3_) *δ_H_* 9.40 (1H, bs, NH), 7.50–7.30 (5H), 7.08 (1H, d, *J* = 15.9 Hz, 4-H), 6.45 (1H, d, *J* = 15.9 Hz, 5-H), 5.45 (1H, bs, NH), 4.25 (2H, q, *J* = 7.1 Hz, OC*H*_2_Me), 1.88 (3H, s, Me), 1.35 (3H, t, *J* = 7.1 Hz, CH_2_*Me*); ^13^C-NMR (100 MHz; CDCl_3_) *δ_C_* 190.2 (C), 169.8 (C), 167.4 (C), 142.1 (CH), 136.8 (CH), 130.5 (CH), 128.9 (CH), 128.4 (CH), 122.6 (CH), 95.5 (C), 60.3 (CH_2_), 26.8 (Me), 14.5 (CH_2_); *m/z* (APcI) 260 (MH^+^, 100%), 243 (12).

#### 3.3.8. (2Z,4E)-2-Amino-3-ethoxycarbonyl-6-phenylhexadien-6-one (**3ab**)

Aminodienone **3ab** was prepared according to the general procedure 3.2.1 using ethyl β-aminocrotonate (**1a**) and 1-phenylprop-2-yn-1-one (**2b**). Purification by column chromatography on SiO_2_ gel, eluting with light petroleum–EtOAc (1:1) (*R_f_* 0.24), gave the title compound as a yellow solid (79 mg, 85%), mp 156–157 ºC (light petroleum–EtOAc) (lit. [4] mp 164 °C) (Found: C, 69.4; H, 6.6; N, 5.2. Calc. for C_15_H_18_NO_3_: C, 69.5; H, 6.6; N, 5.4%) (Found: MH^+^, 260.1282. C_15_H_17_NO_3_ [*MH^+^*] requires 260.1281); IR (KBr)/cm^-1^ *ν_max_* 3,342, 3,203, 2,976, 1,623, 1,580, 1,539, 1,497, 1,378, 1,354, 1,320, 1,286, 1,223, 1,205, 1,178, 1,110, 1,055, 1,036, 1,023, 976, 847, 705, 626; ^1^H-NMR (400 MHz; CDCl_3_) *δ_H_* 9.67 (1H, bs, NH), 7.94 (2H, m, *o*-Ph*H*), 7.86 (1H, d, *J* = 15.0 Hz, 4-H), 7.43 (3H, *m*,*p*-Ph*H*), 7.39 (1H, d, *J* = 15.0 Hz, 5-H), 5.70 (1H, bs, NH), 4.24 (2H, q, *J* = 7.1 Hz, OC*H*_2_Me), 2.33 (3H, s, Me), 1.35 (3H, t, *J* = 7.1 Hz, CH_2_*Me*); ^13^C-NMR (100 MHz; CDCl_3_) *δ_C_* 190.9 (C), 169.8 (C), 166.7 (C), 141.1 (CH), 139.7 (C), 131.8 (CH), 128.4 (CH), 128.1 (CH), 115.8 (CH), 95.6 (C), 60.0 (CH_2_), 22.6 (Me), 14.5 (Me); *m/z* (APcI) 260 (MH^+^, 100%).

#### 3.3.9. (2Z,4E)-2-Amino-3-ethoxycarbonyl-6-(4-chlorophenyl)hexadien-6-one (**3ac**)

Aminodienone **3ac** was prepared according to the general procedure 3.2.1 using ethyl β-aminocrotonate (**1a**) and 1-(4-chlorophenyl)prop-2-yn-1-one (**2c**). Purification by column chromatography on SiO_2_ gel, eluting with light petroleum–EtOAc (1:1) (*R_f_* 0.18), gave the title compound as a yellow solid (91 mg, 86%), m.p. 163–164 ºC (light petroleum–EtOAc) (literature [14] m.p. 164–165 ºC) (Found: MH^+^, 294.0893. C_15_H_17_^35^ClNO_3_ [*MH^+^*] requires 294.0891); IR (KBr)/cm^-1^*ν_max_* 3,315, 3,168, 2,964, 1,653, 1,630, 1,591, 1,571, 1,539, 1,485, 1,350, 1,323, 1,288, 1,224, 1,177, 1,120, 1,089, 1,057, 1,037, 1,012, 975, 856, 824, 743, 713, 642, 589, 538, 502; ^1^H-NMR (400 MHz; CDCl_3_) *δ_H_* 9.71 (1H, bs, NH), 7.88 (1H, d, *J* = 15.0 Hz, 4-H), 7.84 (2H, app d, *J* = 8.5 Hz, 2’,6’-PhH), 7.36 (2H, app d, *J* = 8.5 Hz, 3’,5’-PhH), 7.33 (1H, d, *J* = 15.0 Hz, 5-H), 5.78 (1H, bs, NH), 4.20 (2H, q, *J* = 7.1 Hz, OC*H*_2_Me), 2.27 (3H, s Me), 1.35 (3H, t, *J* = 7.1 Hz, CH_2_*Me*); ^13^C-NMR (100 MHz; CDCl_3_) *δ_C_* 189.5 (C), 169.7 (C), 167.0 (C), 141.6 (CH), 138.0 (C), 138.0 (C), 129.5 (CH), 128.6 (CH), 115.1 (CH), 95.7 (C), 60.1 (CH_2_), 22.6 (Me), 14.5 (Me); *m/z* (APcI) 296 (MH^+^, 32%), 294 (MH^+^, 100).

#### 3.3.10. (2Z,4E)-2-Amino-3-ethoxycarbonyl-6-(4-methoxyphenyl)hexadien-6-one (**3ad**)

Aminodienone **3ad** was prepared according to the general procedure 3.2.1 using ethyl β-aminocrotonate (**1a**) and 1-(4-methoxyphenyl)prop-2-yn-1-one (**2d**). Purification by column chromatography on SiO_2_ gel, eluting with light petroleum–EtOAc (1:1) (*R_f_* 0.50), gave the title compound as a yellow solid (99 mg, 95%), mp 155–156 ºC (light petroleum–EtOAc) (literature [54] m.p. 159 ºC) (Found: MH^+^, 290.1386. C_16_H_20_NO_4_ [*MH^+^*] requires 290.1387); IR (KBr)/cm^-1^ *ν_max_* 3,297, 3,145, 2,974, 1,717, 1,627, 1,598, 1,576, 1,539, 1,511, 1,475, 1,362, 1,321, 1,300, 1,263, 1,231, 1,168, 1,126 1,021, 834, 586; ^1^H-NMR (400 MHz; CDCl_3_) *δ_H_* 9.63 (1H, bs, NH), 7.91 (2H, app d, *J* = 8.8 Hz, 2’,6’-PhH), 7.82 (1H, d, *J* = 15.0 Hz, 4-H), 7.38 (1H, d, *J* = 15.0 Hz, 5-H), 6.85 (1H, app d, *J* = 8.8 Hz, 3’,5’-PhH), 5.63 (1H, bs, NH), 4.23 (2H, q, *J* = 7.2 Hz, OC*H*_2_Me), 3.83 (3H, s, OMe), 2.25 (3H, s Me), 1.35 (3H, t, *J* = 7.2 Hz, CH_2_*Me*); ^13^C-NMR (100 MHz; CDCl_3_) *δ_C_* 189.3 (C), 169.9 (C), 166.3 (C), 162.7 (C), 140.2 (CH), 132.4 (C), 130.2 (CH), 115.7 (CH), 113.6 (CH), 95.5 (C), 60.0 (CH_2_), 55.4 (Me), 22.6 (Me), 14.5 (Me); *m/z* (APcI) 290 (MH^+^, 100%).

#### 3.3.11. (2Z,4E)-2-Amino-3-tert-butoxycarbonylheptadien-6-one (**3ca**)

Aminodienone **3ca** was prepared according to the general procedure 3.2.1 using *tert*-butyl β-aminocrotonate (**1c**) and 4-(trimethylsilyl)but-3-yn-2-one (**2a**). Purification by column chromatography on SiO_2_ gel, eluting with light petroleum–EtOAc (1:1) (*R_f_* 0.26), gave the title compound as a yellow solid (55 mg, 68%), m.p. 142–143 ºC(light petroleum–EtOAc) (literature [14] m.p. 142–144 ºC) (Found: C, 63.8; H, 8.5; N, 6.1. Calc. for C_12_H_19_NO_3_: C, 64.0; H, 8.5; N, 6.2%) (Found: MH^+^, 226.1437. C_12_H_20_NO_3_ [*MH^+^*] requires 226.1438); IR (KBr)/cm^-1^ *ν_max_* 3,341, 3,198, 2,975, 1,654, 1,539, 1,485, 1,454, 1,362, 1,323, 1,295, 1,218, 1,160, 1,111, 1,034, 970, 840, 692, 572; ^1^H-NMR (400 MHz; CDCl_3_) *δ_H_* 9.60 (1H, bs, NH), 7.48 (1H, d, *J* = 15.4 Hz, 4-H), 6.45 (1H, d, *J* = 15.4 Hz, 5-H), 5.39 (1H, bs, NH), 2.20 (3H, s, Me), 2.18 (3H, s, Me), 1.48 (9H, s, CMe_3_); ^13^C-NMR (100 MHz; CDCl_3_) *δ_C_* 198.8 (C), 169.1 (C), 165.2 (C), 139.9 (CH), 120.8 (CH), 95.7 (C), 80.8 (C), 28.5 (Me), 28.4 (Me), 22.7 (Me); *m/z* (APcI) 226 (MH^+^, 98%), 208 (54), and 152 (100).

#### 3.3.12. Ethyl 2,6-dimethylpyridine-3-carboxylate (**4aa**)

Pyridine **4aa** was prepared according to the general procedure 3.2.2 using aminodienone **3aa**. Purification by column chromatography on SiO_2_ gel, eluting with light petroleum–EtOAc (1:1) (*R_f_* 0.44), gave the title compound as a pale yellow oil (24 mg, 66%) (Found: MH^+^, 180.1017. C_10_H_14_NO_2_ [*MH^+^*] requires 180.1019); IR (film)/cm^-1^ *ν_max_* 2,924, 2,825, 1,730, 1,593, 1,462, 1,377, 1,272, 1,236, 1,148, 1,079, 770, 722; ^1^H-NMR (400 MHz; CDCl_3_) *δ_H_* 8.03 (1H, d, *J* = 8.0 Hz, 4-H), 7.00 (1H, d, *J* = 8.0 Hz, 5-H), 4.27 (2H, q, *J* = 7.2 Hz, OC*H*_2_Me), 2.75 (3H, s, Me), 2.50 (3H, s, Me), 1.33 (3H, t, *J* = 7.2 Hz, CH_2_*Me*); ^13^C-NMR (100 MHz; CDCl_3_) *δ_C_* 166.7 (C), 161.1 (C), 159.4 (C), 138.9 (CH), 122.8 (C), 120.5 (CH), 61.1 (CH_2_), 24.8 (Me), 24.6 (Me), 13.7 (Me); *m/z* (APcI) 180 (MH^+^, 100%).

#### 3.3.13. Ethyl 6-methyl-2-phenylpyridine-3-carboxylate (**4ba**)

Pyridine **4ba** was prepared according to the general procedure 3.2.2 using aminodienone **3ba**. Purification by column chromatography on SiO_2_ gel, eluting with light petroleum–EtOAc (1:1) (*R_f_* 0.56), gave the title compound as a pale yellow solid (47 mg, 98%), m.p. 45–46 ºC (EtOH) (literature [55] m.p. 46 ºC) (Found: MH^+^, 242.1175. C_15_H_16_NO_2_ [*MH^+^*] requires 242.1176); IR (film)/cm^-1^ *ν_max_* 3,056, 2,963, 2,915, 2,855, 2,363, 1,715, 1,589, 1,440, 1,356, 1,278 1,211, 1,137, 1,106, 1,053, 797, 767, 740, 699, 650; ^1^H-NMR (400 MHz; CDCl_3_) *δ_H_* 7.95 (1H, d, *J* = 8.0 Hz, 4-H), 7.33–7.25 (5H, PhH), 7.12 (1H, d, *J* = 8.0 Hz, 5-H), 4.05 (2H, q, *J* = 7.1 Hz, OC*H*_2_Me), 2.55 (3H, s, Me), 0.94 (3H, t, *J* = 7.1 Hz, CH_2_*Me*); ^13^C-NMR (100 MHz; CDCl_3_) *δ_C_* 168.2 (C), 160.8 (C), 158.7 (C), 140.5 (C), 138.4 (CH), 128.5 (CH), 128.4 (CH), 128.1 (CH), 124.4 (C), 121.3 (CH), 61.3 (CH_2_), 24.8 (Me), 13.6 (Me); *m/z* (APcI) 242 (MH^+^, 100%).

#### 3.3.14. Ethyl 2-methyl-6-phenylpyridine-3-carboxylate (**4ab**)

Pyridine **4ab** was prepared according to the general procedure 3.2.2 using aminodienone **3ab**. Purification by column chromatography on SiO_2_ gel, eluting with light petroleum–EtOAc (1:1) (*R_f_* 0.62), gave the title compound as a pale yellow solid (47 mg, 98%), m.p. 44–45 ºC (EtOH) (literature [4] m.p. 44 ºC) (Found: MH^+^, 242.1176. C_15_H_16_NO_2_ [*MH^+^*] requires 242.1179); IR (KBr)/cm^-1^ *ν_max_* 2,981, 1,715, 1,582, 1,496, 1,455, 1,382, 1,264, 1,185, 1,154, 1,091, 1,026, 922, 848, 798, 757, 692; ^1^H-NMR (400 MHz; CDCl_3_) *δ_H_* 8.21 (1H, d, *J* = 8.2 Hz, 4-H), 8.02 (2H, m, *o*-Ph*H*), 7.60 (1H, d, *J* = 8.2 Hz, 5-H), 7.42 (3H, *m*,*p*-Ph*H*) 4.32 (2H, q, *J* = 7.2 Hz, OC*H*_2_Me), 2.85 (3H, s, Me), 2.45 (3H, t, *J* = 7.2 Hz, CH_2_*Me*); ^13^C- NMR (100 MHz; CDCl_3_) *δ_C_* 166.9 (C), 160.0 (C), 159.1 (C), 139.3 (CH), 138.5 (C), 129.7 (CH), 128.8 (CH), 127.3 (CH), 123.7 (C), 117.4 (CH), 61.2 (CH_2_), 25.3 (Me), 14.3 (Me); *m/z* (APcI) 242 (MH^+^, 100%).

#### 3.3.15. Ethyl 2-methyl-6-(4-chlorophenyl)pyridine-3-carboxylate (**4ac**)

Pyridine **4ac** was prepared according to the general procedure 3.2.2 using aminodienone **3ac**. Purification by column chromatography on SiO_2_ gel, eluting with light petroleum–EtOAc (1:1) (*R_f_* 0.68), gave the title compound as a pale yellow solid (59 mg, 97%), m.p. 48–49 ºC (aqueous EtOH) (literaute [6] m.p. 47–48 ºC) (Found: MH^+^, 276.0784. C_15_H_15_^35^ClNO_2_ [*MH^+^*] requires 276.0786); IR (KBr)/cm^-1^ *ν_max_* 2,985, 1,720, 1,585, 1,492, 1,455, 1,372, 1,265, 1,180, 1,155, 1,096, 1,013, 896, 833, 786, 742, 705; ^1^H-NMR (400 MHz; CDCl_3_) *δ_H_* 8.23 (1H, d, *J* = 8.2 Hz, 4-H), 7.95 (2H, app d, *J* = 8.5 Hz, 2’,6’-PhH), 7.54 (1H, d, *J* = 8.2 Hz, 5-H), 7.40 (2H, app d, *J* = 8.5 Hz, 3’,5’-PhH), 4.38 (2H, q, *J* = 7.1 Hz, OC*H*_2_Me), 1.60 (3H, s, Me), 1.35 (3H, t, *J* = 7.1 Hz, CH_2_*Me*); ^13^C-NMR (100 MHz; CDCl_3_) *δ_C_* 166.5 (C), 160.1 (C), 157.7 (C), 139.4 (CH), 136.9 (C), 135.9 (C), 129.0 (CH), 128.6 (CH), 123.9 (C), 117.4 (CH), 61.2 (CH_2_), 25.3 (Me), 14.3 (Me); *m/z* (APcI) 278 (MH^+^, 33%), 276 (MH^+^, 100).

#### 3.3.16. Ethyl 2-methyl-6-(4-methoxyphenyl)pyridine-3-carboxylate (**4ad**)

Pyridine **4ad** was prepared according to the general procedure 3.2.2 using aminodienone **3ad**. Purification by column chromatography on SiO_2_ gel, eluting with light petroleum–EtOAc (1:1) (*R_f_* 0.63), gave the title compound as a pale yellow solid (53 mg, 98%), m.p. 68–69 ºC (aqueous EtOH) (literature [6] m.p. 68–69 ºC) (Found: MH^+^, 272.1284. C_16_H_18_NO_3_ [*MH^+^*] requires 272.1281); IR (KBr)/cm^-1^ *ν_max_* 2,989, 2,895, 1,717, 1,605, 1,579, 1,509, 1,452, 1,439, 1,389, 1,362, 1,311, 1,264, 1,172, 1,112, 1,088, 1,071, 1,031, 832, 785; ^1^H-NMR (500 MHz; CDCl_3_) *δ_H_* 8.20 (1H, d, *J* = 8.2 Hz, 4-H), 7.95 (2H, app d, *J* = 8.7 Hz, 2’,6’-PhH), 7.53 (1H, d, *J* = 8.2 Hz, 5-H), 7.37 (2H, app d, *J* = 8.7 Hz, 3’,5’-PhH), 4.34 (2H, q, *J* = 7.1 Hz, OC*H*_2_Me), 3.82 (3H, s, OMe), 2.85 (3H, s, Me), 1.36 (3H, t, *J* = 7.1 Hz, CH_2_*Me*); ^13^C-NMR (125 MHz; CDCl_3_) *δ_C_* 166.8 (C), 161.0 (C), 160.0 (C), 158.7 (C), 139.3 (CH), 131.7 (C), 128.7 (CH), 122.8 (C), 116.5 (CH), 114.2 (CH), 61.1 (CH_2_), 55.4 (Me), 25.4 (Me), 14.4 (Me); *m/z* (APcI) 272 (MH^+^, 100%).

#### 3.3.17. tert-Butyl 2,6-dimethylpyridine-3-carboxylate (**4ca**)

Pyridine **4ca** was prepared according to the general procedure 3.2.2 using aminodienone **3ca**. Purification by column chromatography on SiO_2_ gel, eluting with light petroleum–EtOAc (1:1) (*R_f_* 0.59), gave the title compound [29] as a pale yellow oil (41 mg, 98%) (Found: MH^+^, 208.1329. C_12_H_18_NO_2_ [*MH^+^*] requires 208.1332); IR (film)/cm^-1^ *ν_max_* 2,921, 2,852, 1,724, 1,592, 1,462, 1,377, 1,284, 1,175, 1,144, 1,080, 771, 722; ^1^H-NMR (400 MHz; CDCl_3_) *δ_H_* 7.95 (1H, d, *J* = 8.0 Hz, 4-H), 6.95 (1H, d, *J* = 8.0 Hz, 5-H), 2.68 (3H, s, Me), 2.45 (3H, s, Me), 1.50 (9H, s, CMe_3_); ^13^C-NMR (100 MHz; CDCl_3_) *δ_C_* 166.1 (C), 160.6 (C), 158.7 (C), 138.8 (CH), 124.5 (C), 120.5 (CH), 81.7 (C), 28.2 (Me), 24.8 (Me), 24.6 (Me); *m/z* (APcI) 208 (MH^+^, 100%).

## 4. Conclusions

The use of NIS in EtOH represents a new and rapid mild method for the low temperature cyclodehydration of Bohlmann-Rahtz aminodienone intermediates **3** to give the corresponding 2,3,6-trisubstituted pyridines **4 **in excellent yield and with total regiocontrol. In this process, the NIS appears to be behaving as a remarkable Lewis acid, that catalyzes *E/Z* isomerisation and spontaneous cyclodehydration at 0 ºC, rather than an iodinating agent. This method for mild and efficient cyclodehydration is likely to find application, in particular for the synthesis of pyridines from acid-sensitive substrates, and will be reported [23] in due course.
